# White matter microstructural damage in early treated phenylketonuric patients

**DOI:** 10.1186/s13023-018-0912-5

**Published:** 2018-10-26

**Authors:** María Julieta González, Mónica Rebollo Polo, Pablo Ripollés, Rosa Gassió, Aída Ormazabal, Cristina Sierra, Roser Colomé Roura, Rafael Artuch, Jaume Campistol

**Affiliations:** 1 Neuropediatric Department, PKU Follow Up Unit, Hospital Sant Joan de Déu (HSJD), Institut de Recerca Sant Joan de Deu (IRSJD), Passeig Sant Joan de Deu 2, Postal code, 08950 Barcelona, Spain; 2 Neuroimaging Section, HSJD, IRSJD, Passeig Sant Joan de Deu 2, Postal code, 08950 Barcelona, Spain; 3 Department of Psychology, New York University, 6 Washington Place, 10003 New York, USA; 4 Clinical Biochemistry Department, HSJD, IRSJD, UB, (CIBERER-ISCIII), Passeig Sant Joan de Deu 2, 08950 Barcelona, Spain

**Keywords:** Neuroimaging, Phenylketonuria, Paediatric, Early treatment, Diffusion tensor imaging, Urine monoamines

## Abstract

**Background:**

Despite dietary intervention, individuals with early treated phenylketonuria (ETPKU) could present neurocognitive deficits and white matter (WM) abnormalities. The aim of the present study was to evaluate the microstructural integrity of WM pathways across the whole brain in a cohort of paediatric ETPKU patients compared with healthy controls (HCs), by collecting DTI-MRI (diffusion tensor magnetic resonance imaging) data and diffusion values (mean diffusivity (MD), radial diffusivity (RD) and fractional anisotropy (FA)).

**Methods:**

DTI-MRI data and diffusion values (MD, RD, FA) from WM tracts across the whole brain were analized using Tract Based Spatial Statistics (TBSS), in 15 paediatrics TPKU patients (median age: 12 years) and compared with 11 HCs. Areas showing abnormal values in the patient group were correlated (Pearson) with age, lifetime Phe values, last year median and mean Phe, concurrent Phe values in plasma, urine neurotransmitters status biomarkers, and with a processing speed task.

**Results:**

ETPKU showed bilaterally decreased MD values compared with HCs in the body and splenium of the corpus callosum, superior longitudinal fasciculus, corona radiata and in the posterior limb of the internal capsule. RD values followed a similar pattern, although decreased RD values in PKU patients were also found in the anterior limb of the internal capsule and in the cerebral peduncle. Decreased MD and RD values within the aforementioned regions had significant negative correlations with age, last year median and mean Phe and concurrent Phe values. No correlations were found with monoamines in urine or processing speed task.

**Conclusions:**

ETPKU patients showed MD and RD values significantly decreased across the whole brain when compared with HCs, and this damage was associated with high Phe values and the age of patients. Despite this microstructural damage, no affectation in processing speed was observed in patients with good metabolic control. DTI-MRI sequences could be used as a technique to quantify WM damage that is difficult to be detect in T1 or T2-weighted images, but also to quantify damage of WM through the follow up of patients with poor metabolic control in prospective studies.

## Background

Despite early and continuous dietary intervention, individuals with early treated phenylketonuria (ETPKU) could have neurocognitive deficits and white matter (WM) abnormalities [1]. The aetiology of this process is not entirely understood.

In the last years, diffusion tensor magnetic resonance imaging (DTI-MRI) has emerged as a crucial neuroimaging technique that allows non-invasive assessment of axonal structure and myelin status [2–4]. In particular, by measuring the diffusion of water molecules in the brain, different diffusion indexes can be calculated. Among them, fractional anisotropy (FA), quantifies the preference for diffusion of water molecules in one direction and correlates with axonal diameter, density, and fibre orientation; while mean diffusivity (MD) is related to cellularity, oedema, and necrosis and measures mean water molecular motion [5]. Radial diffusivity (RD) is also very sensitive for the detection of microstructural changes and is usually associated with myelination and axonal diameter [6].

Recent studies have reported abnormal diffusion values (as measured by MD) in the WM of individuals with ETPKU as compared with healthy controls (HCs) [7–15]. Strikingly, the differences in diffusivity extend to regions that showed normal signal intensity when visualised on regular T2-weighted images [7, 15]. Findings regarding the direction of diffusion (as reflected by FA measures) are controversial, with some studies reporting decreased values of diffusion anisotropy in ETPKU [7] while others not finding such differences when compared with control groups [10, 16].

Hallmark neuropathological biomarkers in the brain of both treated and untreated PKU patients are hypomyelination, demyelination or both. Cognitive deficits are present in both treated and untreated PKU individuals, although the link between the neuropathological findings and cognitive deficits is poorly understood. Dyer et al. [17] postulate in this sense two interesting hypothesis. The first is based on the fact that cognitive deficits in individuals with PKU result from a deficiency of the dopamine neurotransmitter (NT). Decreased levels of tyrosine in the PKU brain are thought to be the cause of the low dopamine levels. The second is that elevated phenylalanine (Phe) values inhibit biosynthesis and myelin stability in oligodendrocytes. Myelin influences the maturity of axons, suggesting that axonal maturation may be involved in NT production. Also, abnormalities in the WM matter could be involved in the slower processing speed observed in PKU patients [18].

It has been hypothesized also that the hyperphenylalaninaemia-related neurotoxicity could be caused by a deficiency of large neutral amino acids (LNAA), mainly tyrosine and tryptophan, due to transport competition through the blood-brain barrier (BBB) [19]. Tyrosine and tryptophan are precursors of dopamine and serotonin, respectively; and its relative brain deficit may contribute to a reduced synthesis of these NT but also a disruption in protein synthesis [20–22]. Moreover, high brain Phe values may produce an inhibition of tyrosine and tryptophan hydroxylase activity, causing a further reduction of dopamine and serotonin biosynthesis [23, 24]. Dopamine is essential for proper functioning of the prefrontal cortex (PFC), which governs executive functions. In addition, serotonin is involved both in the cognitive processes mediated by the orbitofrontal cortex and in the regulation of mood, emotions, and behaviour [25].

The aim of the present study was to evaluate the microstructural integrity of WM pathways in a cohort of paediatric ETPKU patients, by collecting DTI-MRI data and extracting FA, RD and MD diffusion values from WM tracts across the whole brain. We first compared ETPKU patient data with a group of HCs and then we correlated the areas showing abnormal values in the patient group with age, last year median and mean Phe, and concurrent Phe values in plasma, lifetime Phe values, urine NT status biomarkers and processing speed task.

## Methods

### Participants

Children with ETPKU (*n* = 15, median age 12 years, range 8–18 years) were recruited through the PKU-Follow-up Unit of Sant Joan de Deu Hospital in Barcelona. All patients were diagnosed by the newborn screening program and were treated continuously from the first weeks of life. Treatment was as follows: eleven with classic dietary control (Phe restriction), five classified with good metabolic control (last year median of Phe values or index dietary control (IDC) [26], was < 360 μmol/L for patients under 12 years old or < 600 μmol/ L after 12 years old), and six with poor metabolic control (IDC was > 360 μmol/L for patients under 12 years old or > 600 μmol/L after 12 years old), according to the European Guidelines [27]. and six with poor metabolic control, IDC was > 360 μmol/L for patients under 12 years old or > 600 μmol/L after 12 years old, according to the European Guidelines [27]. The remaining four patients were treated with tetrahydrobiopterin (BH_4_) and all had good metabolic control. No patients had a history or clinical evidence of neurological deterioration.

### Control group

The control group formed by 11 healthy participants (mean age 11 years old, range 9–18 years; 5 males and 6 females) that were referred for MRI (magnetic resonance imaging) examination for headache. None of the controls had a history of intellectual disability, learning or psychiatric disorders or major medical disorders unrelated to PKU. They had similar age, sex and sociocultural class when compared with the ETPKU group. Controls were included in a consecutive fashion after the consent for the acquisition of the DTI-MRI data was signed.

### Metabolic profile

Concurrent blood Phe (taken the day of the scanning session), lifetime Phe values (calculated as the mean of each median year Phe value across the lifetime), last year median and mean Phe values were measured by ion-exchange chromatography with ninhydrin detection using a Biochrom 30 analyser (Pharmacia-Biotech). Urine excretion of biogenic amine metabolites (homovanillic acid (HVA) for dopamine and 5 hydroxyindoleacetic acid (5HIAA) for serotonin) was analyzed using gas chromatography mass spectrometry detection (Agilent Technologies).

### Neuroimaging

#### Scanning parameters:

A diffusion-weighted MRI (DW-MRI) scanning session was run on a 1.5 T scanner (General Electric Signa HD). Images were acquired with a spin-echo EPI sequence (53 axial slices, TR: 15000 ms, TE: 104 ms, acquisition matrix: 256 × 256, voxel size: 0.94 × 0.94 × 2.5 mm^3^). A run with one non-diffusion weighted volume (using a spin-echo EPI sequence coverage of the whole head) and 25 diffusion-weighted volumes (non-collinear diffusion gradient directions, b-values of 1500 s/mm^2^) was acquired.

#### DTI-MRI preprocessing and statistical analysis:

Diffusion data processing started by correcting for eddy current distortions and head motion using FMRIB’s (functional MRI of the brain) Diffusion Toolbox (FDT), which is part of the FMRIB Software Library [28]. Subsequently, the gradient matrix was rotated to provide a more accurate estimate of diffusion tensor orientations, using FSL’s (FMRIB Software Library) FDT rotating bvecs [29]. Following this, brain extraction was performed using the Brain Extraction Tool [30], which is also part of the FSL distribution. Analysis continued with the reconstruction of the diffusion tensors using the linear least-squares algorithm included in Diffusion Toolkit 0.6.2.2 [31]. Finally, FA, RD and MD maps for each patient and control were calculated using the eigenvalues extracted from the diffusion tensors. Voxel based analyses of FA, RD and MD maps were performed using Tract Based Spatial Statistics (TBSS) [32]. Briefly, FA maps from all individuals were registered to the FMRIB58_FA template (MNI152 space and 1 × 1 × 1 mm^3^) using the nonlinear registration tool (FNIRT) [33]. These registered FA maps were first averaged to create a mean FA volume. Then a mean FA skeleton was derived, which represents the centers of all tracts common to all participants in the study. Each participant’s aligned FA data were then projected onto this skeleton by searching for the highest FA value within a search space perpendicular to each voxel of the mean skeleton. This process was repeated for the RD and MD maps by applying the transformations previously calculated with the FA maps. Finally, in order to assess WM differences between controls and PKU patients, independent sample t-tests were calculated for the RD, MD and FA skeletons, with age and gender as covariates of nuisance. Results were reported with an FWE corrected *p* < 0.05 value using threshold-free cluster enhancement [34] and a nonparametric permutation test with 5000 permutations [35]. Significant voxels within the skeleton were filled to make the presentation of results easier to follow. WM tracts were identified using the JHU-ICBM DTI-81 white matter atlas [36, 37]. RD describes microscopic water movements perpendicular to the axon [38]. It has been proposed to reflect myelin quality along the axon with demyelination being associated with increased RD [39, 40], while MD is more related to tissue density [41]. Finally, for each patient, diffusivity values within the voxels showing significance between group effects were averaged and a mean value was obtained. Pearson’s correlations were computed between these values (which represented individual WM damage) and age, concurrent Phe, last year median, lifetime and mean Phe values, and with HVA and 5HIIA concentrations. Pearson’s correlations were also computed between the diffusivity values and the scores of the neuropsychological tests.

Correlations were computed with MATLAB version R2012a (The MathWorks, Natick, MA, USA). A correlation was considered significant if it survived a *p* < 0.05 false discovery rate (FDR) corrected threshold.

Given that the 15 ETPKU patients can be subdivided into 2 different groups (good metabolic control and poor metabolic control) we completed one last analysis. Again, using the average RD/MD (no significant results were obtained for FA, see next section) values from all voxels showing differences between patients and controls (same values used for the correlational analyses described above), we tested whether the different ETPKU subgroups showed different percentages of reduction in diffusivity values (i.e., WM damage). Taking into account the reduced number of patients per group, we used nonparametric tests under SPSS (version 18.0.0) to perform these calculations.

### Neuropsychological evaluation

The Wechsler Intellectual Scale of Children (WISC-IV) [43] and Wechsler Adults Intelligence Scale (WAIS-III) [44] were administered to assessment general intellectual ability (intellectual quotient (IQ)). Given that WM abnormalities have been associated with a slower performance in processing speed, the following neuropsychological battery was used to evaluate it: Processing Speed Index of Wechsler Scales, time required to copy the Rey Complex Figure Task [45], motor and visual search speed with Trail Making Test Part A [46], speed naming with Speeded Naming NEPSY (NEuroPSYchological Assessment) II subtest [47], response speed with hit reaction time of Conners’ Continuous Performance Test-II (CPT-II) [48] and Total initiation time (sum of time taken to begin each item) in execution of Tower of London test [49].

The reference typical punctuation for IQ, processing speed in Index Scales Wechsler, time required to copy the Rey Complex Figure Task (RCFT), speed naming in NEPSY II, Trail Making Test A and total initiation time in Tower of London, were 100 ± 15. The reference typical punctuation in the evaluation of hit reaction time in CPT-II: Conners’ Continuous Performance Test-II, was 50 ± 10, as it was considered faster execution less than 40 and slower execution more than 60.

### Systematic review

The literature published from 2001 to 2016 were systematically searched: in PubMed: http://www.ncbi.nlm.nih.gov/pubmed. To avoid any risk of bias, general search terms were chosen: clinical studies, PKU/phenylketonuria, DTI/diffusion tensor imaging, magnetic resonance imaging, early treated, late treated, paediatric and adults.

Inclusion criteria: studies in humans with control group, neuroimaging studies of DTI done in paediatric or adult PKU patients, early or late treated. Exclusion criteria: isolated case reports, studies in animal/cellular models, and patients with other metabolic disease such as BH4 deficiencies, articles not published in English. A total of 12 articles met criteria, 9 of them studied only early treated PKU (described in Table 1) [7–15], while the other articles described late and early treated PKU patients compared with a healthy control group (described in Table 2) [16, 50, 51].

**Table 1:** Neuroimaging studies with revision samples only ETPKU

Authors	Studied population	Studied regions	Parameters of DTI	Conclusions
Vermathen et al. 2007 [7]	ETPKU adult patients (mean age 32.5 years) (*n* = 9). Control group (mean age 29.4 years) (*n* = 7).	Grey and white matter tracts. Include corpus callosum (CC)	MD, FA	Decreased MD and FA values in lesions and CC. Decreased MD and FA values correlated negatively with Phe values.
White et al. 2010 [8]	ETPKU paediatric patients (mean age 12.2 years) (*n* = 34). Control group (mean age 12.4 years) (*n* = 61).	6 ROI (region of interest) of CC (genu, rostral body, anterior midbody, posterior midbody, isthmus and splenium)	MD, RA = FA	Decreased MD values in anterior part of CC. Non significant differences in FA compared to control group. Age related decrement of anterior WM of CC. Non-significant correlations with MD and Phe values.
White et al. 2013 [9]	ETPKU adults and paediatric patients BH4 responders (mean age 18.2 years) (*n* = 12). Control group (mean age 17.8 years) (*n* = 9).	10 ROI. Include CC (genu, body and splenium)	MD	Basal MD values improve after 6 months with BH4 treatment. MD values correlate negatively with Phe levels.
Atenor-Dorsey et al. 2013 [10]	ETPKU adults and paediatric patients (mean age 18 years) (*n* = 29). Control group (mean age 17.8 years) (*n* = 12)	10 ROI. Include CC (genu, body and splenium)	MD, FA	Decreased MD values correlated with poor executive functions. Decreased MD values compared to control group. Non significant differences in FA values compared to control group.
Peng et al. 2014 [11]	ETPKU adult and paediatric patients (mean age 23.3 years) (*n* = 10). Control group (mean age 23.5 years) (*n* = 12).	12 ROI. Include CC (genu, body and splenium)	MD, RD, AD, FA	Decreased MD, RD and AD values in WM tracts and CC compared to control group. Decreased MD, RD and AD values correlated with older ETPKU. Non-significant differences in FA compared to control group.
Wesonga et al. 2016 [12]	ETPKU paediatric patients (mean age 12.2 years) (*n* = 31). Control group (mean age = 12 years) (*n* = 51).	10 ROI. Include CC (genu, body and splenium)	MD	Age correlated with decreased MD values in 4 out of 10 ROI.
Hood et al. 2015 [13]	ETPKU paediatric patients (mean age 12.2 years) (*n* = 36). Control group (*n* = 24).	Over 10 ROI. Include CC (genu, body and splenium)	MD	Decreased MD values were correlated with high exposure of Phe levels.
Hood et al. 2016 [14]	ETPKU paediatric patients (mean age 12.2 years) (*n* = 36). Control group (*n* = 62).	2 ROI: PPO (posterior parietal-occipital) CSO (centrum semiovale).	MD, RD, FA	Decreased MD and RD values compared to control group. Non significant differences in FA compared to control group.
Ding et al. 2008 [15]	Adult patients (range 17–32 years): ETPKU (*n* = 4). Control group (*n* = 4).	22 ROI. Include CC (corpus and splenium).	MD, FA	Decreased MD values in WM and GM (grey matter) than control group. FA non significant differences than control group.

**Table 2:** Neuroimaging studies with early (ETPKU) and late treated PKU (LTPKU)

Authors	Studied population	Studied regions	Parameters of DTI	Conclusions
Leuzzi et al. 2007 [16]	Adult and paediatric patients (*n* = 32): ETPKU (*n* = 21) (mean age 17.1 years), LTPKU (*n* = 11) (mean age 22.4 years. Control group (*n* = 30) (mean age 32.9 years) (range: 12–58 years).	4 ROI: Parietal (P), Occipital (O), Frontal (F), Temporal (T).	MD, FA.	Abnormal signal T2-W and FLAIR scans: Parietal periventricular WM abnormalities > O > F > T. WM severity score correlated with age patient at time of the study. MD values and WM scores were closely and inversely correlated (*p* < .001). Blood and brain Phe levels were closely correlated (*p* < .001). Brain Phe levels was unrelated with FA values.
Kono et al. 2005 [50]	Adult and paediatric patients (*n* = 21) (mean age 19.4 years) (age range 3–44 years): ETPKU (*n* = 14), LTPKU (*n* = 7). Control group (*n* = 21) (mean age 20.6 years) (age range 3–33 years).	6–10 ROI (anterior and posterior deep WM).	MD	MD values in posterior deep WM significantly lower than in frontal deep WM (*P* < .001). MD values in the posterior WM tended to be lower with increased concurrent serum Phe levels (*p* < .005) and average serum Phe last year of examination (*p* < .001).
Scarabino et al. 2009 [51]	Adult and paediatric patients (*n* = 32) (mean age 18.9 years): ETPKU (*n* = 21), LTPKU (*n* = 11). Control group (*n* = 30) (mean age 32.9 years).	4 ROI: (P, O, F, T).	MD, FA	Supratentorial (periventricular and subcortical) abnormal T2: P > O > F > T. Decreased 30–50% of MD compared to control group. FA non correlated with Phe values.

## Results

The 15 ETPKU evaluated patients had a concurrent Phe mean value of 426 μmol/L (range: 102–1162), lifetime Phe values of 319.5 μmol/L (range: 151–560), a last year Phe median value of 397 μmol/L (range: 134–852), a last year mean value of 440 μmol/L (range: 186–837). The scores for the intellectual quotient (IQ; WISC-IV and WAIS-III) ranged from 95 to 116 (mean: 111). The individual clinical, biochemical and molecular features of the PKU patients were described in Table 3. The results of neuropsychological evaluation were described in Table 4. Only 3 patients had a slower processing speed (more than one altered task), and all were patients with poor metabolic control.

**Table 3:** Clinical characteristics of patients

Patient code	Sex	Age	Mutation	IDC	Concurrent Phe μmol/L	Last year median Phe umol/L	Last year mean Phe umol/L	Lifetime mean Phe umol/L
P1	Female	8 years	R158G/L48S	Good	258	256.5	263	268
P2	Female	8.9 years	Y206X/L348 V	Good	435	330.5	298	434
P3	Male	13.6 years	R261Q-R176X	Good	503	558.5	597.7	319
P11	Female	8 years	I65T/IVS12 + 1A > G	Good	126	134	186.8	151
P12	Female	8 years	R261Q-I65T	Good	443	285	288.5	297
P6	Male	12 years	delF39/F55 L	Good (BH4)	667	434	398	316
P7	Female	13.1 years	R241Q- Not found	Good (BH4)	330	369.5	383	309
P8	Female	13.7 years	Y414C/K396 M	Good (BH4)	376	384	407	334
P9	Female	17.1 years	V388 M-P362T	Good (BH4)	585	410.5	522	322
P4	Male	14.6 years	IVS8nt-7a > g-/IVS8nt + 1g > a	Poor	1162	801.5	789	560
P5	Male	17.8 years	I65T-R261X	Poor	1016	852	837.4	406
P10	Female	8 years	IVS4 + 5G > T / IVS4 + 5G>	Poor	417	442.5	436	351
P13	Male	9.1years	IVS10-IVS10	Poor	567	372	440.5	320
P14	Male	9.3 years	IVS4 + 5G > T/ IVS10	Poor	102	426	366.5	285
P15	Male	10.7 years	R158Q/P281S	Poor	198	427	390.7	367

**Table 4:** Results of neuropsychological evaluation

Patient code	Index dietary control (IDC)	IQ^a^	Processing speed Index^a^	RCFT^a^ (Time required to copy)	CPT-II^b^ (Hit reaction time)	NEPSY II^a^ (Naming speed)	Trail Making Test A^a^	Tower of London^a^ (Initiation time)
P1	Good	105	110	92	40	95	106	98
P2	Good	95	88	96	58	75	88	116
P3	Good	114	93	94	45	95	103	130
P11	Good	114	115	100	65	80	83	104
P12	Good	113	117	106	61	84	108	120
P6	Good (BH4)	109	99	69	59	95	93	98
P7	Good (BH4)	115	112	90	40	100	113	106
P8	Good (BH4)	115	121	90	51	95	96	122
P9	Good (BH4)	97	102	102	57	–	103	94
P4	Poor	95	85	58	68	84	115	96
P5	Poor	116	107	81	52	–	94	100
P10	Poor	101	115	126	55	95	97	112
P13	Poor	102	99	106	62	84	104	102
P14	Poor	114	91	119	69	75	96	112
P15	Poor	113	91	73	46	80	73	102

*IQ* intellectual quotient, *RCFT* The Rey Complex Figure Task, *CPT-II* Conners’ Continuous Performance Test- II

^a^Typical Punctuation (TP): 100 ± 15

^b^TP: 50 ± 10. Fast: < 40; Slow: > 60

### Parameters of DTI: RD, MD and FA

**Fig. 1:**
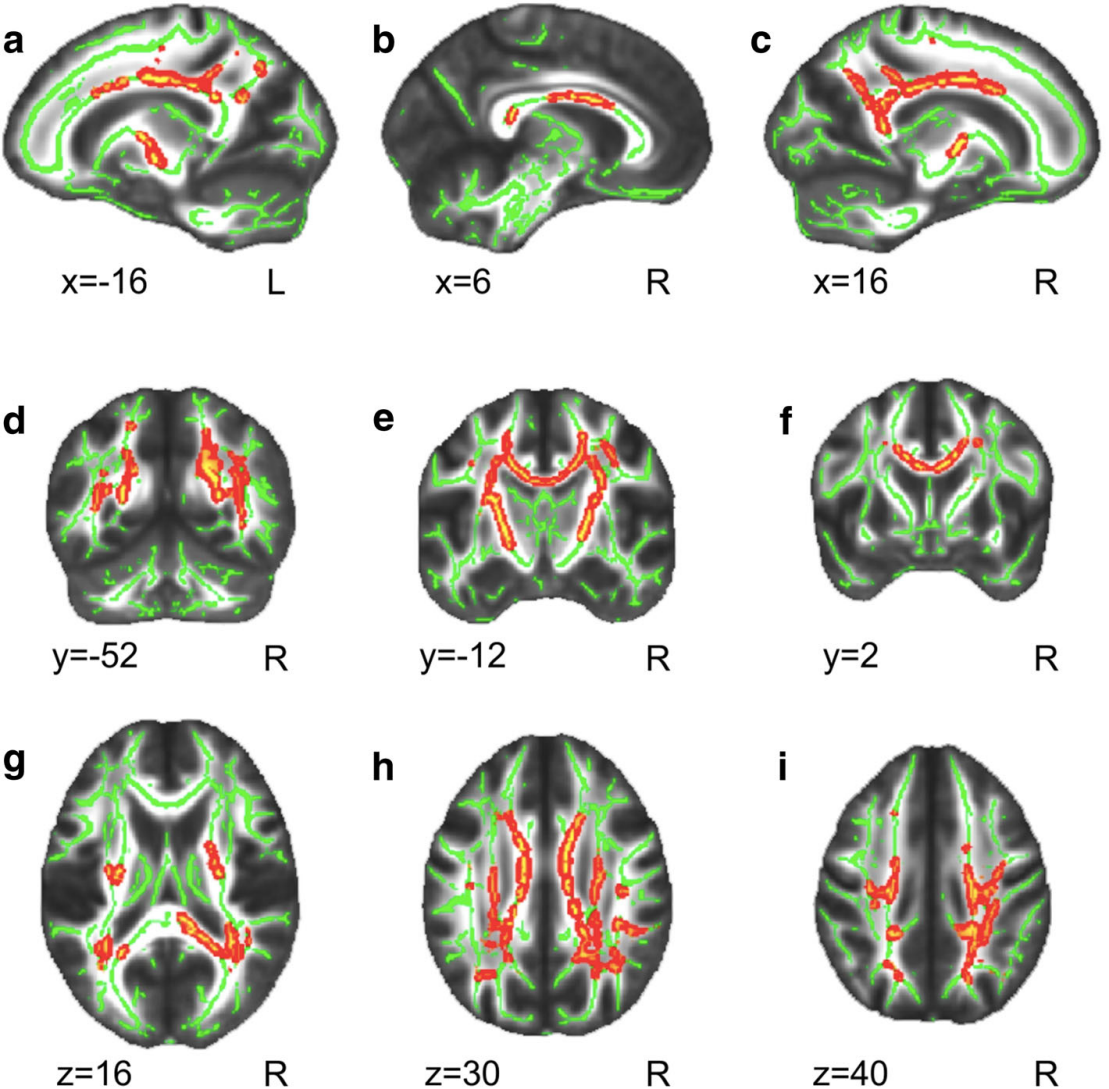
Decreased Mean Diffusivity values of WM tracts across the whole brain in PKU as compared with controls. **a-i**: Results are shown over the mean group skeleton (in green), which represents the centers of all WM tracts common to all participants in the study (see Materials and Methods). In red-yellow, the WM regions showing decreased MD in patients as compared with controls are shown (*p* < 0.05 FWE corrected). Neurological convention is used with MNI (Montreal Neurological Institute) coordinates at the left bottom of each slice

**Fig. 2:**
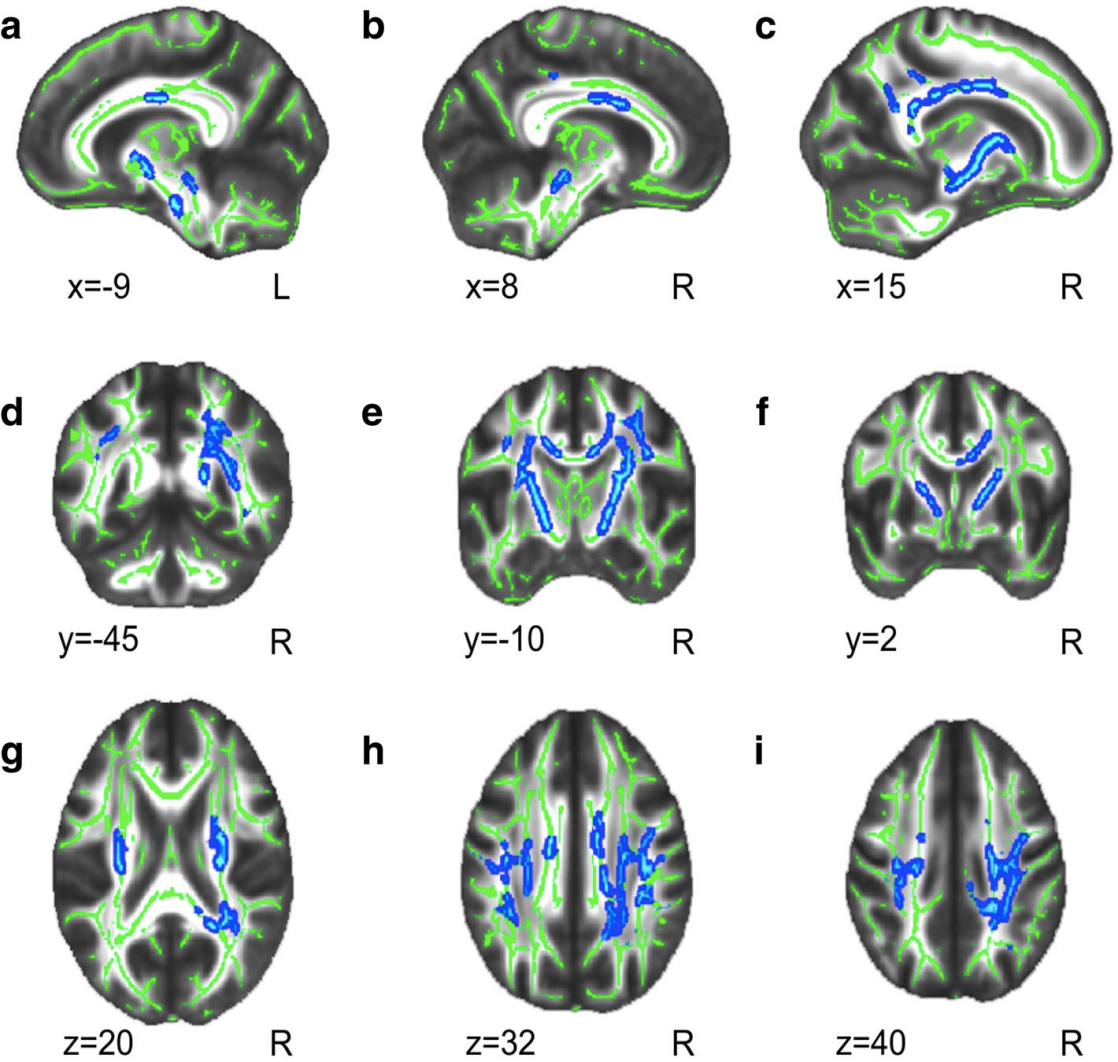
Decreased Radial Diffusivity values of WM tracts acress the whole brain in PKU as compared with controls. **a-i**: Results are shown over the mean group skeleton (in green), which represents the centers of all WM tracts common to all participants in the study (see Materials and Methods). In blue, the WM regions showing decreased RD in patients as compared with controls are shown (p < 0.05 FWE corrected). Neurological convention is used with MNI (Montreal Neurological Institute) coordinates at the left bottom of each slice

In spite of the diffusion data coming from normal appearing white matter, eleven patients had subtle periventricular abnormalities in a T2 sequence, with only one being more evident and extended. However, only three had normal T2 sequences in MRI. Accordingly, the whole ETPKU group showed decreased MD values when compared with controls bilaterally in the body and splenium of the corpus callosum (CC), superior longitudinal fasciculus, corona radiata and in the posterior limb of the internal capsule (Fig. 1). RD values followed a very similar pattern, although decreased RD values in ETPKU patients were also found in the anterior limb of the internal capsule and in the cerebral peduncle (Fig. 2). FA values showed no significant differences between groups.

### Associations among age, full scale IQ, processing speed task, blood Phe values and urinary neurotransmitter biomarkers

**Fig. 3:**
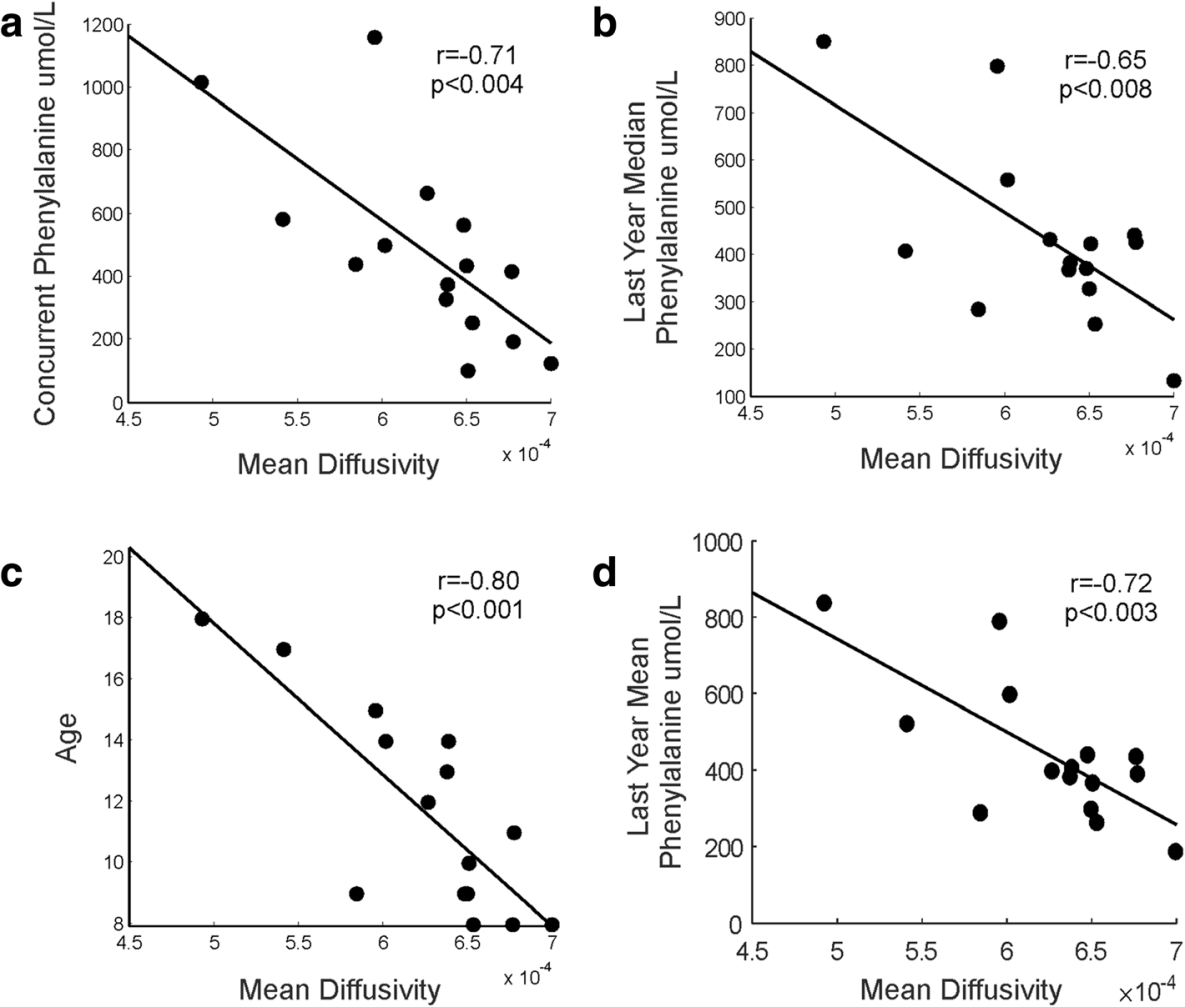
Correlations between the average Mean Diffusivity value of the WM pathways showing between-group differences and biochemical parameters and age. The scatter plots display: **a** The correlation between the mean MD value of the voxels and concurrent Phe. **b** The correlation between the mean MD value of the voxels and last year median. **c** The correlation between the mean MD value of the voxels and age. **d** The correlation between the mean MD value of the voxels and last year mean. The scatter plots display the correlation between the mean MD value of the voxels showing significant differences and concurrent Phe, last year median and mean Phe values and age (the greater the age/phenylalanine values the greater the reduction in MD)

**Fig. 4:**
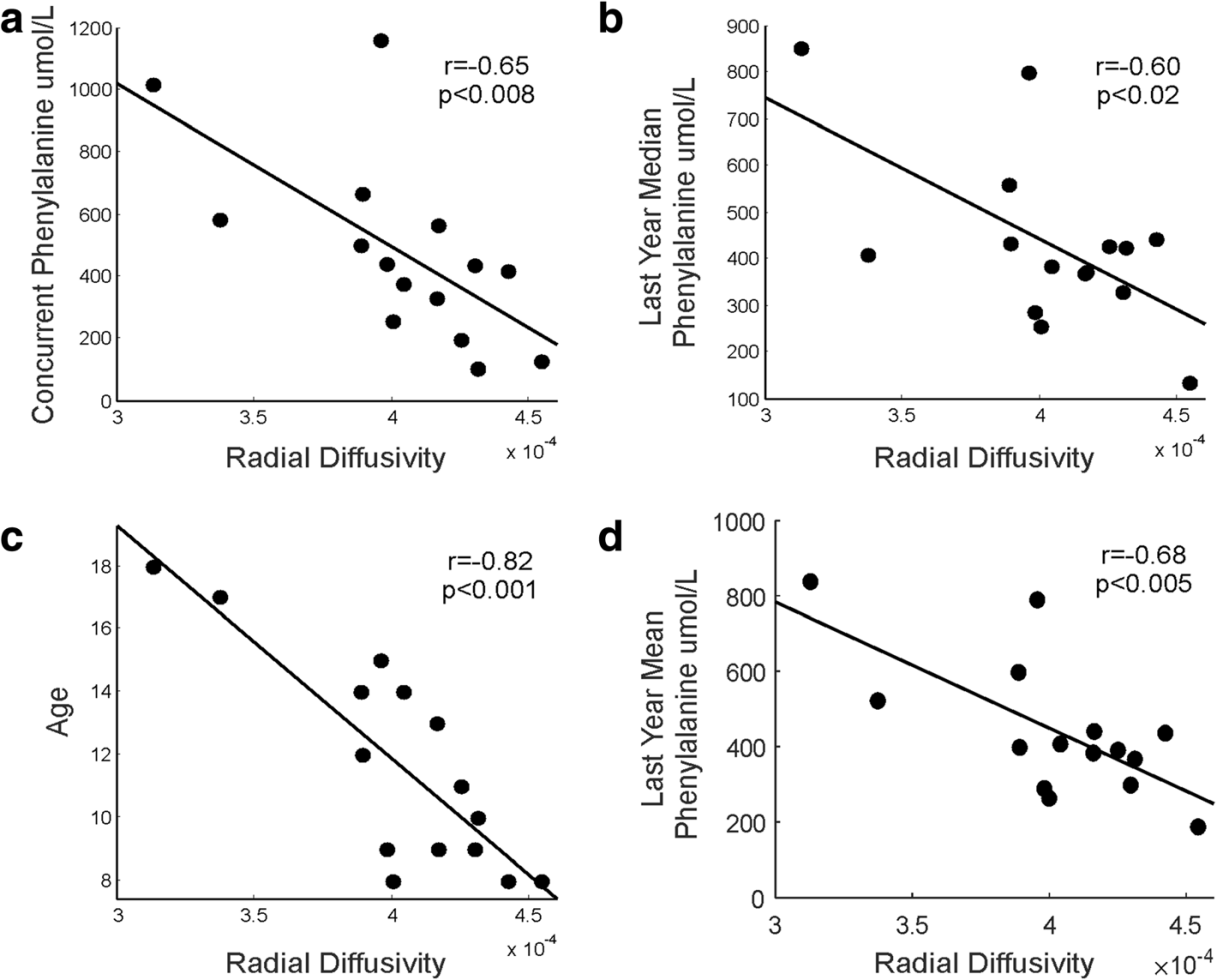
Correlations between the average Radial Diffusivity value of the WM pathways showing between-group differences and biochemical parameters and age. The scatter plots display: **a** The correlation between the mean RD value of the voxels and concurrent Phe. **b** The correlation between the mean RD value of the voxels and last year median. **c** The correlation between the mean RD value of the voxels and age. **d** The correlation between the mean RD value of the voxels and last year mean. The scatter plots display the correlation between the mean RD value of the voxels showing significant differences and concurrent Phe, last year median and mean Phe values and age (the greater the age/phenylalanine values the greater the reduction in RD)

Average MD values for all voxels showing WM damage, significantly and negatively correlated with age (*r* = − 0.80, *p* < 0.001), last year median Phe, last year mean Phe and concurrent Phe values (*r* = − 0.65, *p* < 0.008, *r* = − 0.72, *p* < 0.003 and *r* = − 0.71, *p* < 0.004, respectively; all correlations survived a *p* < 0.05 FDR corrected threshold; Fig. 3). RD values followed the same pattern as MD values, also significantly and negatively correlating with age (*r* = − 0.82, p < 0.001), last year median Phe, last year mean Phe and concurrent Phe values (*r* = − 0.60, *p* < 0.02, *r* = − 0.68, *p* < 0.005, r = − 0.65, p < 0.008, respectively; all correlations survived a *p* < 0.05 FDR corrected threshold; Fig. 4).

Thus, the greater the age or the higher the median, mean and concurrent Phe values, the greater the reduction in MD and RD values. This suggests that increased WM damage (reduced MD and RD values) is related to higher concentrations of Phe and that this damage increases with age.

All patients had lifetime Phe values in normal range in recomended levels for age: Lifetime Phe values were less than 600 μmol/l in patients older than 12 years old and less than 360 umol/L in the younger group (< 12 years old). These values fall within the recomended normal ranges for each age reported in the European PKU guidelines [42]. No significant correlations were observed between lifetime Phe values and MD or RD variables.

While correlations were not significant between diffusion values and HVA (MD: *r* = 0.28, *p* = 0.30; RD: *r* = 0.30, *p* = 0.27), the relationship between 5HIAA and MD (*r* = 0.47, *p* = 0.073), and also RD (*r* = 0.49, *p* = 0.068), approached significance. There were no significant correlations between IQ, and processing speed scores and DTI-MRI variables. No significant differences were found between the PKU-sub-groups regarding the diffusivity values.

## Discussion

This work evaluated the microstructural integrity of WM pathways across the whole brain in a sample of paediatric ETPKU patients. By means of DTI-MRI parameters such as FA, MD and RD and using TBSS, we compared WM tracts across the whole brain of ETPKU patients as compared to controls. We found that ETPKU patients showed bilaterally decreased MD values compared with HCs in the body and splenium of the CC, superior longitudinal fasciculus, corona radiata and in the posterior limb of the internal capsule. These findings were consistent with previous observations [7–15]. RD values also followed a similar pattern as previous studies [11, 14].

The most frequent findings in neuroimaging studies in these patients are the presence of WM abnormalities, evidenced as an increased signal intensity in T2-weighted sequences [52–54], with the periventricular WM being the most commonly affected region [55–58]. Only 3 (20%) patients showed normal T2 in our sample. The extent and severity of the WM abnormalities appears to be modulated by patient age, dietary adherence and metabolic control profile of Phe levels [54, 57, 59]. More recent studies demonstrate that DTI-MRI can provide additional insight into the microstructure of WM integrity of ETPKU patients. Indeed, previous findings have shown decreased MD values in ETPKU patients [8–11, 50].

Reviewing previous studies, only two of them studied MD, RD and FA in a paediatric population in more than one brain region. Peng and colleagues [11] studied a smaller series of patients than the present work (but mixed both paediatric and adult patients), and its results agree with those of this study. Hood and colleagues [14] studied a larger series, comparable to our study, which also showed a decrease in MD and RD values in paediatric ETPKU patients, but their analyses were restricted only to the posterior parietal occipital (PPO) and centrum semiovale (CSO; i.e., they did not study WM tracts across the whole brain). Both studies showed non-significant differences in FA values when compared with the control group. A brief revision among the different PKU neuroimaging studies is summarized in Table 1 [7–15] and Table 2 [16, 50, 51].

Associations between decreased MD and Phe values have previously been reported [7, 11–13]. In our study, we demonstrate that last year median and mean Phe and concurrent Phe values were significantly correlated with decreased MD and RD values.

In addition, decreased MD within the CC has also been shown to be related to Phe levels [11, 13] . White et al. [8] showed MD restriction values in the anterior part of the CC (genu and rostral body). Wesonga et al. [12] demonstrated significant abnormal diffusion values in the genu and splenium of the CC. In contrast, we found decreased of MD values but also RD decreased values in the body and splenium of the CC.

Finally, decreased MD values have been previously shown to correlate with increased age [8, 11, 12] in PKU children, which further supports the results shown in this work. MD and RD are thought to be a good marker of myelin structure and, in healthy populations, decreased RD and MD values are usually associated to a better WM microstructure [2, 40, 60, 61]. Moreover, research shows that in healthy populations MD values decrease with age during childhood (increased myelination) while WM is still maturating [62], whereas during adulthood, MD increases with age (decreased myelination) as WM degenerates [63, 64]. However, PKU populations including the one studied in this work usually display reduced MD and RD values that are related to increased Phe levels [11, 13]. That is, in PKU patients reduced RD and MD values, rather than reflecting improved WM structure, are a proxy of WM damage (note that elevated Phe values inhibit biosynthesis and myelin stability in oligodendrocytes). Thus, it could be that in healthy populations the negative association between MD and RD values and age reflects increased myelination [62], while in PKU, the same relationship could suggest quite the opposite: we hypothesize that high levels of Phe produce an accumulative damage in the microstructure of WM.

For future prospective studies, it is important to note that to identify the damaged white matter pathways affected in PKU patients, it is paramount to create and use age specific DTI templates from healthy participants. While these templates could be obtained from existing atlases, we suggest that, to avoid inter-scanner effects, it would be useful to have a specific template made for each scanner and center. In addition, these templates could be used to compare the microstructural white matter lesions of each individual patient against the control group of the hospital, by means of, for example, Crawford-Howell t-tests [65, 66]; for an example of this test in single-patient DTI data, see Tuomiranta et al. [67].

While we did not find a significant correlation between diffusivity values and HVA, the relationship between 5HIAA and WM damage approached significance. Although it is known that dopamine and serotonin status may be affected in PKU patients, urinary HVA and 5-HIAA are, apparently, not good biomarkers for evaluation. Our results further support that blood Phe level is still the most reliable biomarker in the follow-up of these patients.

In addition, previous studies suggest that slower processing speed is related to the WM damage usually found in PKU, as a result of disruptions in the speed with which neural signals are transmitted [18, 68]. Nevertheless, despite the microstructural damage, not affectation in processing speed was observed in this group of patients. An explanation could be that the PKU population recruited for this study had in general a good metabolic control. Possibly higher Phe values would have led to more extensive WM abnormalities than the ones showed by this group of ETPKU patients, and this, in turn, could have been manifested in a slower performance in processing speed.

## Conclusions

When we evaluated the WM tracts across the whole brain using TBSS in paediatric ETPKU patients, we found MD and RD values significantly decreased compared with HCs, and this damage was associated with high Phe values and with the age of patients. No correlations were found with processing speed scores.

Given current and previous research, we recommend the use of DTI-MRI sequences for the neuroimaging study of PKU patients. This technique could be used not only to quantify WM damage that is difficult to detect in T1 or T2-weighted images (MRI sequences routinely used), but also to quantify damage of WM through the follow up of poor metabolic control patients in prospective studies.

In addition, we suggest that neuropsychological evaluation should be performed routinely in patients with good metabolic control after 7 years old, in particular in individuals with WM abnormalities.

## Abbreviations

5-HIAA: 5 - hydroxyindolacetic acid
BBB: Blood brain barrier
BH_4_: Tetrahydrobiopterin
CC: Corpus callosum
CSO: Centrum semiovale
DTI-MRI: Diffusion tensor magnetic resonance imaging
DW-MRI: Diffusion-weighted MRI
ETPKU: Early treated phenylketonuria
FA: Fractional anisotropy
FDR: False discovery rate
FDT: Diffusion Toolbox
FMRIB’s: Functional MRI of the brain
FNLRT: Functional Non Linear Registration Tool
FSL’s: Functional MRI of the brain Software Library
HCs: Healthy controls
HVA: Homovanillic acid
IDC: Index dietary control
IEC: Independent ethics committees
IQ: Intellectual quotient
LTPKU: Late treated phenylketonuria
LNAAs: Large neutral amino acids
MATLAB: Matrix laboratory
MD: Mean diffusivity
MRI: Magnetic resonance imaging
NEPSY II: Developmental NEuroPSYchological Assessment version II
NT: Neurotransmitter
PFC: Prefrontal cortex
Phe: Phenylalanine
PKU: Phenylketonuria
PPO: Posterior parietal occipital
RD: Radial difusivity
ROI: Region of interest
TBSS: Tract Based Spatial Statistics
WAIS-III: Wechsler Adults Intelligence Scale version –III
WISC-IV: Wechsler Intellectual Scale of Children version IV
WM: White matter

## References

[1] Blau N, van Spronsen FJ, Levy HL (2010). Phenylketonuria. Lancet.

[2] Basser PJ, Mattiello J, LeBihan D (1994). Estimation of the effective self-diffusion tensor from the NMR spin echo. J Magn Reson B.

[3] Hüppi PS, Dubois J (2006). Diffusion tensor imaging of brain development. Semin Fetal Neonatal Med.

[4] Yoshida S, Oishi K, Faria AV, Mori S (2013). Diffusion tensor imaging of normal brain development. Pediatr Radiol.

[5] Sen PN, Basser PJ (2005). A model for diffusion in white matter in the brain. Biophys J.

[6] Alexander AL, Hurley SA, Samsonov AA, Adluru N, Hosseinbor AP, Mossahebi P (2011). Characterization of cerebral white matter properties using quantitative magnetic resonance imaging stains. Brain Connect.

[7] Vermathen P, Robert-Tissot L, Pietz J, Lutz T, Boesch C, Kreis R (2007). Characterization of white matter alterations in phenylketonuria by magnetic resonance relaxometry and diffusion tensor imaging. Magn Reson Med.

[8] White DA, Connor LT, Nardos B, Shimony JS, Archer R, Snyder AZ (2010). Age-related decline in the microstructural integrity of white matter in children with early- and continuously-treated PKU: a DTI study of the corpus callosum. Mol Genet Metab.

[9] White DA, Antenor-Dorsey JA, Grange DK, Hershey T, Rutlin J, Shimony JS (2013). White matter integrity and executive abilities following treatment with tetrahydrobiopterin (BH4) in individuals with phenylketonuria. Mol Genet Metab.

[10] Antenor-Dorsey JA, Hershey T, Rutlin J, Shimony JS, McKinstry RC, Grange DK (2013). White matter integrity and executive abilities in individuals with phenylketonuria. Mol Genet Metab.

[11] Peng H, Peck D, White DA, Christ SE (2014). Tract-based evaluation of white matter damage in individuals with early-treated phenylketonuria. J Inherit Metab Dis.

[12] Wesonga E, Shimony JS, Rutlin J, Grange DK, White DA (2016). Relationship between age and white matter integrity in children with phenylketonuria. Mol Genet Metab Rep.

[13] Hood A, Antenor-Dorsey JA, Rutlin J, Hershey T, Shimony JS, McKinstry RC (2015). Prolonged exposure to high and variable phenylalanine levels over the lifetime predicts brain white matter integrity in children with phenylketonuria. Mol Genet Metab.

[14] Hood A, Rutlin J, Shimony JS, Grange DK, White DA (2016). Brain white matter integrity mediates the relationship between phenylalanine control and executive abilities in children with phenylketonuria. JIMD Rep.

[15] Ding XQ, Fiehler J, Kohlschütter B, Wittkugel O, Grzyska U, Zeumer H (2008). MRI abnormalities in normal-appearing brain tissue of treated adult PKU patients. J Magn Reson Imaging.

[16] Leuzzi V, Tosetti M, Montanaro D, Carducci C, Artiola C, Carducci C (2007). The pathogenesis of the white matter abnormalities in phenylketonuria. A multimodal 3.0 tesla MRI and magnetic resonance spectroscopy (1H MRS) study. J Inherit Metab Dis.

[17] Dyer CA (1999). Pathophysiology of phenylketonuria. Ment Retard Dev Disabil Res Rev.

[18] Janos AL, Grange DK, Steiner RD, White DA (2012). Processing speed and executive abilities in children with phenylketonuria. Neuropsychology.

[19] Pardridge WM (2015). Blood-brain barrier endogenous transporters as therapeutic targets: a new model for small molecule CNS drug discovery. Expert Opin Ther Targets.

[20] Güttler F, Lou H (1986). Dietary problems of phenylketonuria: effect on CNS transmitters and their possible role in behaviour and neuropsychological function. J Inherit Metab Dis.

[21] Ribas GS, Sitta A, Wajner M, Vargas CR (2011). Oxidative stress in phenylketonuria: what is the evidence?. Cell Mol Neurobiol.

[22] González MJ, Gassió R, Artuch R, Campistol J (2016). Impaired neurotransmission in early-treated pnhenylketonuria patients. Semin Pediatr Neurol.

[23] Van Spronsen FJ, Hoeksma M, Reijngoud DJ (2009). Brain dysfunction in phenylketonuria: is phenylalanine toxicity the only possible cause?. J Inherit Metab Dis.

[24] De Groot MJ, Hoeksma M, Blau N, Reijngoud DJ, van Spronsen FJ (2010). Pathogenesis of cognitive dysfunction in phenylketonuria: review of hypotheses. Mol Genet Metab.

[25] Grace AA, Gerfen CR, Aston-Jones G (1998). Catecholamines in the central nervous system: overview. Adv Pharmacol.

[26] Vilaseca MA, Campistol J, Cambra FJ, Lambruschini N (1995). Index of dietary control of PKU patients. Quím Clin.

[27] Jenkinson M, Beckmann CF, Behrens TE, Woolrich MW, Smith SM (2012). FSL. Neuroimage.

[28] Leemans A, Jones DK (2009). The B-matrix must be rotated when correcting for subject motion in DTI data. Magn Reson Med.

[29] Smith SM (2002). Fast robust automated brain extraction. Hum Brain Mapp.

[30] Wang R, Wedeen VJ. Athinoula A. Martinos Center for Biomedical Imaging, Department of Radiology, Massachusetts General Hospital. Development of TrackVis is funded by MGHGCRC and NIMH Grant 5R01MH064044. http://trackvis.org/dtk/

[31] Smith SM, Jenkinson M, Johansen-Berg H, Rueckert D, Nichols TE, Mackay CE (2006). Tract-based spatial statistics: voxelwise analysis of multi-subject diffusion data. Neuroimage.

[32] Andersson JLR, Jenkinson M, Smith SM. Non-linear registration, aka spatial normalisation. In: FMRIB technical report TR07JA2; 2007. http://www.fmrib.ox.ac.uk/analysis/techrep/tr07ja2/tr07ja2.pdf.

[33] Smith SM, Nichols TE (2009). Threshold-free cluster enhancement: addressing problems of smoothing, threshold dependence and localisation in cluster inference. NeuroImage.

[34] Nichols TE, Holmes AP (2002). Nonparametric permutation tests for functional neuroimaging: a primer with examples. Hum Brain Mapp.

[35] Mori Susumu, Wakana Setsu, Nagae-Poetscher Lidia M., Van Zijl Peter C.M. (2005). Introduction. MRI Atlas of Human White Matter.

[36] Hua K, Zhang J, Wakana S, Jiang H, Li X, Reich DS (2008). Tract probability maps in stereotaxic spaces: analyses of white matter anatomy and tract-specific quantification. NeuroImage.

[37] Klawiter EC, Schmidt RE, Trinkaus K, Liang HF, Budde MD, Naismith RT (2011). Radial diffusivity predicts demyelination in ex vivo multiple sclerosis spinal cords. NeuroImage.

[38] Fields RD (2008). White matter in learning, cognition and psychiatric disorders. Trends Neurosci.

[39] Zatorre RJ, Fields RD, Johansen-Berg H (2012). Plasticity in gray and white: neuroimaging changes in brain structure during learning. Nat Neurosci.

[40] Song SK, Sun SW, Ramsbottom MJ, Chang C, Russell J, Cross AH (2002). Dysmyelination revealed through MRI as increased radial (but unchanged axial) diffusion of water. NeuroImage.

[41] Sagi Y, Tavor I, Hofstetter S, Tzur-Moryosef S, Blumenfeld-Katzir T, Assaf Y (2012). Learning in the fast lane: new insights into neuroplasticity. Neuron.

[42] Van Spronsen FJ, van Wegberg AM, Ahring K, Bélanger-Quintana A, Blau N, Bosch AM (2017). Key European guidelines for the diagnosis and management of patients with phenylketonuria. Lancet Diabetes Endocrinol.

[43] Wechsler D (2007). Wechsler intelligence scale for children. 4th ed. (WISC-IV).

[44] Wechsler D (1999). Wechsler intelligence scale for adults.

[45] Rey A (2003). Rey-Osterrieth complex figure test.

[46] Strauss E, Sherman EMS, Spreen O, Strauss E, Sherman EMS, Spreen O (2006). Trail Making Test. A compendium of neuropsychological tests. Administration, norms, and commentary.

[47] Korkman M, Kirk U, Kemp S (2014). NEPSY-II.

[48] Conners CK. Conners' Continuous Performance Test II. Version five for Windows (CPT II. V.5). Madrid: Pearson Clinical; 2004; https://www.pearsonclinical.co.uk/Psychology/ChildMentalHealth/ChildADDADHDBehaviour/ConnersContinuousPerformanceTestIIVersion5forWindows(CPTIIV5)/PDFReports/Profile.pdf.

[49] Culbertson W, Zillmer E (2005). Tower of London- Drexel University TOLDX.

[50] Kono K, Okano Y, Nakayama K, Hase Y, Minamikawa S, Ozawa N (2005). Diffusion-weighted MR imaging in patients with phenylketonuria: relationship between serum phenylalanine levels and ADC values in cerebral white matter. Radiology.

[51] Scarabino T, Popolizio T, Tosetti M, Montanaro D, Giannatempo GM, Terlizzi R (2009). Phenylketonuria: white-matter changes assessed by 3.0-T magnetic resonance imaging (MR), MR spectroscopy and MR diffusion. Radiol Med.

[52] Villasana D, Butler IJ, Williams JC, Roongta SM (1989). Neurological deterioration in adult phenylketonuria. J Inherit Metab Dis.

[53] Thompson AJ, Smith I, Brenton D, Youl BD, Rylance G, Davidson DC (1990). Neurological deterioration in young adults with phenylketonuria. Lancet.

[54] Thompson AJ, Tillotson S, Smith I, Kendall B, Moore SG, Brenton DP (1993). Brain MRI Changes in phenylketonuria. Associations with dietary status. Brain.

[55] Bick U, Fahrendorf G, Ludolph AC, Vassallo P, Weglage J, Ullrich K (1991). Disturbed myelination in patients with treated hyperphenylalaninaemia: evaluation with magnetic resonance imaging. Eur J Pediatr.

[56] Bick U, Ullrich K, Stöber U, Möller H, Schuierer G, Ludolph A (1993). White matter abnormalities in patients with treated hyperphenylalaninaemia: magnetic resonance relaxometry and proton spectroscopy findings. Eur J Pediatr.

[57] Cleary M, Walter J, Wraith J, Jenkins J, Alani S, Tyler K (1994). Magnetic resonance imaging of the brain in phenylketonuria. Lancet.

[58] Phillips M, McGraw P, Lowe MJ, Mathews VP, Hainline BE (2001). Diffusion-weighted imaging of white matter abnormalities in patients with phenylketonuria. Am J Neuroradiol.

[59] Anderson PJ, Wood SJ, Francis DE, Coleman L, Warwick L, Casanelia S (2004). Neuropsychological functioning in children with early-treated phenylketonuria: impact of white matter abnormalities. Dev Med Child Neurol.

[60] Basser PJ (1995). Inferring microstructural features and the physiological state of tissues from diffusion-weighted images. NMR Biomed.

[61] Song SK, Yoshino J, Le TQ, Lin SJ, Sun S, Cross AH (2005). Demyelination increases radial diffusivity in corpus callosum of mouse brain. Neuroimage.

[62] Scantlebury N, Cunningham T, Dockstader C, Laughlin S, Gaetz W, Rockel C (2014). Relations between white matter maturation and reaction time in childhood. J Int Neuropsychol Soc.

[63] Madden DJ, Spaniol J, Costello MC, Bucur B, White LE, Cabeza R (2009). Cerebral white matter integrity mediates adult age differences in cognitive performance. J Cogn Neurosci.

[64] Benitez A, Jensen JH, Falangola MF, Nietert PJ, Helpern JA (2018). Modeling white matter tract integrity in aging with diffusional kurtosis imaging. Neurobiol Aging.

[65] Crawford JR, Howell DC (1998). Regression equations in clinical neuropsychology: an evaluation of statistical methods for comparing predicted and obtained scores. J Clin Exp Neuropsychol.

[66] Crawford JR, Garthwaite PH (2012). Single-case research in neuropsychology: a comparison of five forms of t-test for comparing a case to controls. Cortex.

[67] Tuomiranta LM, Camara E, Froudist WS, Ripolles P, Saunavaara JP, Parkkola R (2014). Hidden word learning capacity through orthography in aphasia. Cortex.

[68] Anderson PJ, Wood SJ, Francis DE, Coleman L, Anderson V, Boneh A (2007). Are neuropsychological impairments in children with early-treated phenylketonuria (PKU) related to white matter abnormalities or elevated phenylalanine levels?. Dev Neuropsychol.

